# Observations on Rheumatism, and on Burning of the Feet

**Published:** 1836-10-01

**Authors:** 


### Observations on Rheumatism. By Mr. *Malcolmson.*

In a volume of essays lately published by Mr. M. at the expense of the Madras Government, we find some very curious observations on rheumatism, as it exists amongst the natives, and also the Europeans, in our oriental possessions. It appears that, excepting fever, no disease is more prevalent among sepoys than rheumatism ; and that, though seldom fatal, it causes the loss of more men to the service than all other diseases put together. As it is often confounded with Beriberi (a dangerous and frequently mortal malady), it is probably much more fatal than is supposed. It rarely presents itself in the acute form among the natives—and then does not differ from the same complaint in Europe.

"But these acute and subacute forms of rheumatism are of little practical importance compared to the common chronic affection, which, whether succeeding the acute, or arising without evident cause, is a disease of great obstinacy, little under the influence of medicine, and lays the foundation of numerous constitutional ailments. When it attacks a healthy person, it may at first be confined to a single joint, which is followed by the affection of others, generally without any relief to the one first suffering. The pain is not generally very acute nor always worse at night, and often is little more than an aching and weakness of the joint." 7.

The parts most frequently affected are in the following order—the knees, ankles, heels, elbows, wrists, hips, shoulders, back and chest. The pain in the knees is always much complained of, and is generally referred to the whole joint—sometimes to one condyle. There is no heat or swelling—and the patient is often suspected of skulking and sent on duty, till swelling takes place, with general bad health, when the mistake is discovered. The heels suffer severely, the pain extending up the tendo Achillis, with swelling about its insertion.

"The soles of the feet, the calves of the legs or flesh of the thighs suffer more rarely than the loins or hips. The roots of the toes, the tarsal ligaments, the back of the hands and joints of the fingers are frequently swollen and painful ; and the muscular parts of the back, the sides of the chest, the ribs and the sternum often become painful, tender, and swell. And in many rheumatic cases, where the patients were ascertained to have had no venereal complaint for years, nor to have taken mercury, the shin bones have suffered from pain, and even from swelling. The tissues, then, that appear most generally involved in the disease are the ligamentous and tendinous, the periosteum, and more rarely the muscular, and we shall see reason to believe that many others are occasionally engaged." 8.

In a large proportion of protracted cases, the patient loses flesh and strength, and becomes cachectic. European treatment is rarely applicable. Many get well by rest and frictions. This last measure is necessary in all forms of the disease, and is the chief means of cure, with the exception of mercury, which is, "beyond comparison, the best remedy in Indian rheumatism."

### Burning of the Feet.

This curious complaint has only come into notice since the Burmese war, and very little has been written on the subject. It seems that an ignorance of this disease has led to the sacrifice of many lives, and entailed great expense on the Government.

"In a disease like this, of which nothing was known, and which had no sensible signs by which it could be recognised; when the patient was not emaciated, as often happened in elderly men of a naturally corpulent habit, and in slight cases amongst the young, he was suspected of malingering to avoid harassing duties or to procure leave to return to his own country; an opinion which would naturally lead to the greatest practical cruelty, in refusing rest in hospital, leave of absence and change of climate to men, to whom they afforded the only chance of recovery." 34.

Melancholy instances of this kind have come to our author's knowledge, where men have died in making exertions beyond their strength, while being treated as malingerers!! This led to want of confidence on the part of the natives, and even the Europeans, in the medical officers. Our author does not consider this disease as a sequela of Indian rheumatism, as it is unknown in places where the latter complaint is most prevalent. The following case and description of the disease will give the reader some idea of this singular malady.

*Case.* "A paria prisoner, æt. 24, admitted 1st Jan. came to hospital yesterday complaining of sense of numbness of his body and a burning of his feet, with costive bowels. Pulse at that time frequent and skin was slightly warm. Had half a drachm of compound powder of jalap which operated four times; says to day that the numbness is better, but the burning of the feet, and he now adds of the abdomen, is no better; pulse 120, not very strong, skin is natural, tongue pretty clean; in walking moves steadily enough, except that he does not place his feet at once firmly on the ground, and he complains of pain in the calves of the legs. Has been ill 3 days. To take treeak farook pills and a diet of meat and wheat cakes. 2d. Pulse to day 112, skin natural; says he feels better of the heat in his feet and abdomen, and that the numbness is quite gone, bowels twice opened. 3d. Pulse 106, skin natural, one stool; says he has no complaint. 4th. Pulse 96, no heat or numbness. 7th. Pulse 84; no complaint. Omitt. med. 9th. Discharged.

This case was evidently allied to beriberi, which was prevailing at the time, and of which burning in the feet is an occasional symptom. It does not, however, often occur in that disease, and is found to affect the soles and the calves of the legs, the back of each side of the spine and occasionally the flesh of the legs, in all of which a connexion can be traced with other affections of the nerves of the part. The symptom is not confined to any class of cases, being observed in recent and slight examples, and in old and hopeless ones, but in which I have in general, found the numbness not very great, and to be combined with other signs of slightly obstructed nervous power. Both, then, appear to depend on an affection of the nerves, and their occurring together would suggest that they are modifications of the same disease, which is further supported, by the fatal cases exhibiting many of the graver symptoms of beriberi. The evidence of an occasional correspondence in some of the symptoms, and even of the appearances on dissection, are however by no means sufficient to identify two diseases, as these must often run into each other, if similar parts are affected. The general history of the complaints, therefore, affords the only satisfactory evidence, on which I am enabled to communicate the result of extensive observation, in the most favourable circumstances which have ever presented themselves. On the return of the troops from Ava in the middle of 1826, the corps that were sent to the northern division were in general suffering from 'burning of the feet,'

but no beriberi shewed itself till some months after, when the other complaint had almost entirely disappeared; and after this, and the disappearance of the sloughing ulcers and bowel-complaints connected with depraved habit which prevailed with them, these regiments were very healthy, until the period of residence and season disposed them to the endemic of the circars. It was also observed, that men with burning of the feet were not peculiarly liable to beriberi, which attacked indiscriminately all new comers, whether from Rangoon or from places in India, although it was true that those men whose constitutions were impaired by this or other disease on foreign service, were rather more liable both to beriberi and fever. This observation was not confined to any particular corps, but was equally true of all those suffering from 'burning of the feet,' which entered the districts where beriberi prevailed, and also when stationed in towns where other troops were losing men from it. It was perhaps more instructively exhibited in the 4th extra regiment at Ellore than in any other. This corps was raised in January, 1826, and was composed of recruits and of men returned sick from Rangoon, who were transferred to it. Amongst the latter, burning of the feet prevailed, but did not attack any of the other men, and before beriberi made its appearance, which did not happen till the end of the year, when the subjects of it had been exposed to the influence of the place for many months, the former disease had disappeared, except in a few obstinate cases, none of which ran into beriberi. The first instances of beriberi were not of a decided character. A man suffering from fever having lost the use of his limbs in September, and in October a sickly boy had œdema of the limbs and face with swelled belly, but without any affection of the nerves. These facts, observed as they were on the large scale in both diseases, afforded much better evidence of the pathological difference of these complaints, than can be derived from a similarity in the latter stages, with some resemblance in the appearances on dissection."

"'Burning of the feet,' which is the designation usually applied by Europeans, from the distressing sensation being for the most part confined to the soles, is merely a translation of the ordinary expression of a native soldier on presenting himself at the hospital, and has no pretensions to accuracy, but it deserves to be retained, as marking the distinction and importance of the affection, till a more accurate knowledge is acquired of its nature. The burning frequently extends over the surface of the lower extremities, which are affected with severe pains, in many instances confined to the fleshy parts, especially the soles of the feet and calves, as in other cases of affection of the nerves at their origin, and the limbs sooner or later emaciate. The hands have partaken of the morbid state, and in a few cases the burning has extended to the whole body and even to the face. The parts are dry and do not feel hot to the touch, but I made no observations with the thermometer; and on this subject, and the distribution of the morbid sensations, and their relations to other symptoms often present, no accurate observations have been made. In some cases the burning is stated to be worse at night, in which it partakes of the nature of those more common sensations which arise from slight nervous derangements common in dyspepsy, menstrual irregularity, and convalescence from severe disease. Nor is there any reason to doubt, that nervous irritability from deranged capillary circulation was occasionally a cause of the morbid sensation in Ava, as it unquestionably was of the analogous complaint in the prisoner, in whom it was removed by the diaphoretic action of Dover's powder, which, conjoined with tonics, Good states to be the proper remedy for sensations of this kind depending on irritable habit.*

* "A gentleman of robust habit, but subject to slight disorder of the bowels, was much distressed with painful scalding sensations in the soles and palms, coming on in the evening and lasting most of the night. After some time, the

Besides the pains in the lower extremities and emaciation, symptoms of a generally depraved habit were present; and in the worst cases extensive organic disease. The skin was dry and harsh, often scaly or covered with itch, the patient was harassed by irregular attacks of fever; he felt weak and was exhausted on slight exertion, to which he often had a great aversion. The tongue was usually pale, swollen, smooth or furred, and only red when the intestinal mucous membrane was excited. The gums were observed by several gentlemen to be swollen and soft, but the symptom was neither common nor remarkable in degree. Night blindness was not of unfrequent occurrence as in some forms of scurvy. Cough was more common than in any other complaint to which the natives are liable, and there was distressing dyspnœa in the advanced cases. The pulse was little altered in the early stages, and, when uninfluenced by the organic affections, was small, irritable, and easily excited by exertion or irritation of any kind. The digestion was in almost every instance impaired; the appetite was weak and irregular, and the most wholesome food caused uneasiness at stomach, puffy belly or pain. The abdomen was often tympanitic and tender to the touch; diarrhœas, dysentery, pain in the course of the colon or around the umbilicus frequently occurred, and increased the emaciation or destroyed the patient. In some instances, however, both the appetite, digestion, and evacuations were natural. In the worst cases, stiffness and numbness of the lower extremities were complained of. Dropsical swellings of the legs were not uncommon, and usually the result of debility alone, disappearing on the patient being enabled to keep in the recumbent posture, but also in many instances the effect of organic changes, and complicated with effusion into the cavities. Although in most cases where 'burning of the feet' was complained of, careful enquiry could detect signs of general disorder in the tongue, skin or abdomen, a few had no other symptom, and yet the disorder was real, and in time caused emaciation and bad health. In some all the general symptoms and even pain in the calves and soles of feet, numbness, &c. have been present without burning; and in others, the pains in the limbs have left the patient, and yet the burning was unrelieved many months after the patient's general health was restored, by his return to his own country. Nor is this surprising, when the different kinds of morbid sensations induced by even local disease of nerves are considered. Neither will the appearances on dissection throw any *certain* light on the cause of the sensation, the same morbid changes having taken place where this symptom was wanting as where it was present; but they point to the lower part of the spine as the seat of the local disorder, and are of the greatest use in leading us to a correct estimate of the formidable nature of symptoms, which end in extensive alterations in the structure of the viscera of all the cavities. It is of great importance, however, to observe, that these alterations are not essential to the disease, as a very large proportion of the patients, if placed in favorable circumstances, may be restored to health in a moderate period of time." 43.

Of the treatment little can be said. The essential object is change of climate, and a return to the usual habits of life and food in the sepoy, with exemption from hard duty.

### Rheumatism of Europeans in India.

This has been almost entirely neglected by tropical writers. It has al-

---

parts became tender to the touch, so that walking was painful, and the skin exhibited spots of a reddish tint which were slightly elevated. He was dyspeptic and the skin was usually dry. He derived temporary benefit from bathing the feet in hot water, and from blue-pill and James's powder; but he owed his recovery to active exercise, by which a free perspiration was excited."

ready been observed that rheumatic fever is rare in India, and requires, when it occurs, less active treatment than in colder climes. The tartrite of antimony has proved very serviceable in these cases.

But the chronic rheumatism of the East is a more local affection than in the West, where it is often dependent on, or connected with, constitutional disorder. In the East, it assimilates with neuralgia, and is a very frequent sequela of intermittents. In short, there is little doubt that, in many cases, it is the product of malaria, affecting the whole constitution or a part of it. But, it may be asked, how *a part?* Our answer is, that we have strong suspicion that the exposure of a limb, for example, to malarious exhalations, especially in the night, will induce rheumatism, independently of cold or moisture. When these last accompany malaria, the cause is of course augmented in intensity.

" In feverish districts rheumatism is very prevalent, partly from the moisture and variable climate being exciting causes of both diseases, and partly from the frequent origin of rheumatism in long-continued attacks of intermittent and remittent fever." 58.

Our author thinks that the Indian rheumatism is often caused by the abuse of mercury—which, if used with prudence, is the best remedy.

" Notwitstanding my conviction of the frequency of mercurial rheumatism, I can assert on the ground of extensive experience, that mercury in small doses and judicious combination is the most generally effectual remedy in all forms of rheumatism in Europeans in India; and the remarks on its employment in natives apply without any material modification to Europeans. It is especially necessary to give it in small doses, for very little will affect the mouth, and as there is danger of relapse being caused by the peculiar effect of mercury, it should never be given more frequently or continued longer than is absolutely necessary. This caution is the more necessary, as it is stated in some recent works, that mercury affects the system with difficulty in acute rheumatism; and in other cases, ten grains of calomel every night, or five grains two or three times a day are recommended, which is far more than I consider safe. Mr. Wyllie, formerly cantonment-surgeon at Nagpore, who was averse to the free employment of calomel in other diseases, assured me, that it was the only agent which had any power over the severe rheumatism of that place." 68.

Mr. Malcolmson adverts to another cause of pains in external parts—namely, disorder of the hepatic system.

" In some severe cases of hepatitis the soreness of the skin, bones, and muscles extends over the whole right side of the chest, and gives to the disease much of the appearance of severe pleuritic inflammation, or rheumatism of the chest. In such circumstances, after free evacuations the pain and ordinary symptoms of hepatitis become more distinct, and the diffused pain and tenderness leave the patient. I have found this external tenderness to the slightest touch the *only early general symptom of fatal thoracic disease;* it is therefore necessary to be on our guard in such cases, and to observe their progress with more closeness, than the observations of the professor would lead us to think necessary." 76.

We must here conclude our short notice of the work, for which, by the way, we have to return our thanks to the Medical Board of Madras, who were kind enough to present it to us.

